# Galeterone and The Next Generation Galeterone Analogs, VNPP414 and VNPP433-3β Exert Potent Therapeutic Effects in Castration-/Drug-Resistant Prostate Cancer Preclinical Models In Vitro and In Vivo

**DOI:** 10.3390/cancers11111637

**Published:** 2019-10-24

**Authors:** Andrew K. Kwegyir-Afful, Senthilmurugan Ramalingam, Vidya P. Ramamurthy, Puranik Purushottamachar, Francis N. Murigi, Tadas S. Vasaitis, Weiliang Huang, Maureen A. Kane, Yuji Zhang, Nicholas Ambulos, Sudhir Tiwari, Pratima Srivastava, Ivo P. Nnane, Arif Hussain, Yun Qiu, David J. Weber, Vincent C. O. Njar

**Affiliations:** 1 Department of Pharmacology, University of Maryland School of Medicine, 685 West Baltimore Street, Baltimore, MD 21201, USA; Andrew.kwegyir-Afful@fda.hhs.gov (A.K.K.-A.); rv.darsini@gmail.com (V.P.R.); PPuranik@som.umaryland.edu (P.P.); njamurigi@gmail.com (F.N.M.); YQiu@som.umaryland.edu (Y.Q.); 2 Center for Biomolecular Therapeutics, University of Maryland School of Medicine, 685 West Baltimore Street, Baltimore, MD 21201, USA; DWeber@som.umaryland.edu; 3 Department of Pharmaceutical Sciences, School of Pharmacy and Health Professions, University of Maryland Eastern Shore, 207 Somerset Hall, Princess Anne, MD 21853, USA; tsvasaitis@umes.edu; 4 Department of Pharmaceutical Sciences, University of Maryland School of Pharmacy, Baltimore, MD 21201, USA; whuang@rx.umaryland.edu (W.H.); mkane@rx.umaryland.edu (M.A.K.); 5 Division of Biostatistics and Bioinformatics, University of Maryland Marlene and Stewart Greenebaum Comprehensive Cancer Center, Baltimore, MD 21201, USA; Yuzhang@som.umaryland.edu; 6 Department of Epidemiology and Public Health, University of Maryland School of Medicine, Baltimore, MD 21201, USA; 7 Department of Microbiology and Immunology and University of Maryland Marlene and Stewart Greenebaum Comprehensive Cancer Center, Baltimore, MD 21201, USA; NAmbulos@som.umaryland.edu; 8 DMPK (Biology Division), GVK BIO, Nacharam, Hyderabad 500076, India; Sudhir.Tiwari@gvkbio.com (S.T.); pratima.srivastava@gvkbio.com (P.S.); 9 Janssen Research & Development, Spring House, PA 19477, USA; nnanepalle@hotmail.com; 10 Veterans Affairs Medical Center, Baltimore, MD 21201, USA; AHussain@som.umaryland.edu; 11 Marlene and Stewart Greenebaum Comprehensive Cancer Center, University of Maryland School of Medicine, 685 West Baltimore Street, Baltimore, MD 21201, USA; 12 Department of Biochemistry and Molecular Biology, University of Maryland School of Medicine, Baltimore, MD 21201, USA

**Keywords:** prostate cancer, castration-/drug-resistant PC cell, galeterone (Gal), NGGAs, VNPP433-3β AR/AR-V7, Mnk1/2 degraders, Mnk-eIF4E/mTORC1 signaling pathways, apoptosis

## Abstract

These studies compared the efficacies of our clinical agent galeterone (Gal) and the FDA-approved prostate cancer drug, enzalutamide (ENZ) with two lead next generation galeterone analogs (NGGAs), VNPP414 and VNPP433-3β, using prostate cancer (PC) in vitro and in vivo models. Antitumor activities of orally administered agents were also assessed in CWR22Rv1 tumor-bearing mice. We demonstrated that Gal and NGGAs degraded AR/AR-V7 and Mnk1/2; blocked cell cycle progression and proliferation of human PC cells; induced apoptosis; inhibited cell migration, invasion, and putative stem cell markers; and reversed the expression of epithelial-to-mesenchymal transition (EMT). In addition, Gal/NGGAs (alone or in combination) also inhibited the growth of ENZ-, docetaxel-, and mitoxantrone-resistant human PC cell lines. The NGGAs exhibited improved pharmacokinetic profiles over Gal in mice. Importantly, in vivo testing showed that VNPP433-3β (at 7.53-fold lower equimolar dose than Gal) markedly suppressed (84% vs. Gal, 47%; *p* < 0.01) the growth of castration-resistant PC (CRPC) CWR22Rv1 xenograft tumors, with no apparent host toxicity. ENZ was ineffective in this CRPC xenograft model. In summary, our findings show that targeting AR/AR-V7 and Mnk1/2 for degradation represents an effective therapeutic strategy for PC/CRPC treatment and supports further development of VNPP433-3β towards clinical investigation.

## 1. Introduction

**Figure 1:**
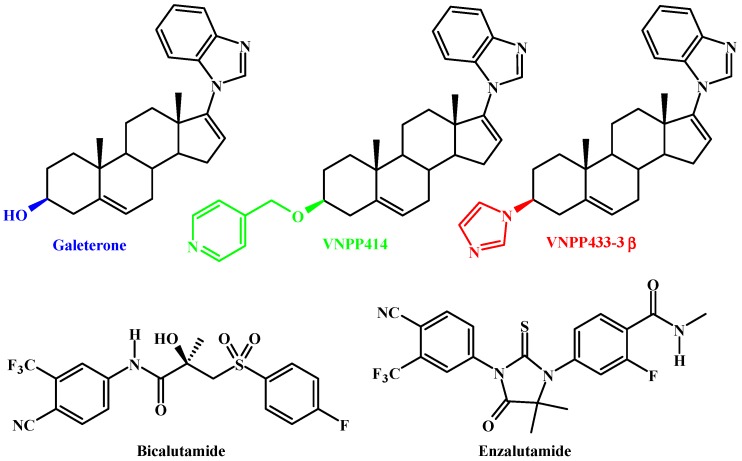
Chemical structures of Galeterone, VNPP414 and VNPP433-3β, Bicalutamide, and Enzalutamide.

Galeterone (Gal; Figure 1), a steroidal molecule designed by our group to inhibit 17α-hydroxylase/17.20-lyase (CYP17), a key enzyme in the steroidogenic pathway that lie at the crossroads of androgen and corticoid biosynthesis was found to disrupt the androgen receptor (AR) signaling pathway via inhibition of androgen synthesis (CYP 17 inhibition; reviewed in [1]), AR antagonism, and AR degradation [1,2,3]. Gal progressed successfully through Phase 1 and Phase 2 clinical trials in men with castration-resistant prostate cancer (CRPC) but failed in the pivotal phase 3 clinical trials [4,5,6]. It is relevant to note that Educational & Scientific LLC (ESL), Baltimore, USA, announced (17 December 2018) that the University of Maryland, Baltimore (UMB) has granted ESL an exclusive license for the development of Gal for the treatment of prostate cancer [7]. We look forward to the initiation and outcome of the new phase III clinical trials of Gal in men with prostate cancer.

In studies designed to enhance the anticancer efficacy of Gal, we discovered potent anti-prostate cancer Gal analogs via structural modification of the C-3 hydroxy group [3,8]. We also discovered that Gal and the next generation Gal analogs (NGGAs), in addition to modulation of AR signaling, also effectively target oncogenic eukaryotic protein translation, via suppression of the cap-dependent protein translational complex, mitogen-activated protein kinase-interacting kinases 1 and 2 (Mnk1/2)-eukaryotic initiation factor 4E (eIF4E) signaling. Mechanistically, we have established that modulation of the Mnk1/2-eIF4E signaling is via Gal/NGGAs-induced ubiquitin-proteasomal degradation of Mnk1/2 proteins [9,10]. Thus, these unique small molecules are also Mnk1/2 degraders (MNKDs) [11]. It is noteworthy that Mnk1/2 are the only kinases known to drive phosphorylation of eIF4E on Ser209 in vivo [12,13,14], to promote its tumorigenic potential [11,15,16,17,18].

In recent studies, D’Abronzo and colleagues discovered that elevated phosphorylated eIF4E (p-eIF4E) levels induced by antiandrogen bicalutamide (Figure 1) rendered prostate cancer cells in tumor xenografts and clinical tumors resistant to antiandrogen/mTORC1-inhibitor treatment [19], suggesting that targeting of Mnk1/2 signaling may be a critical adjunct for effective treatment of prostate cancer [9,11,20,21,22]. Furthermore, upregulation of Mnk1/2-eIF4E signaling has also been implicated in the development of drug resistance in a variety of human cancers [9,11,16,20,21,22,23,24,25,26,27]. As Mnk1/2-eIF4E signaling is implicated in cancer development, progression, metastasis and drug-resistance, we therefore sought to determine the effects of Gal and two lead NGGAs AR/AR-V7 and Mnk1/2 degraders, VNPP414 and VNPP433-3β (Figure 1) in several drug-naïve and drug/castration-resistant prostate cancer cell lines and xenografts. Of note, the FDA-approved 2nd generation antiandrogen (AR inhibitor), enzalutamide (ENZ; Figure 1), is reported to stimulate eIF4E(S209) phosphorylation and prevent further treatment with combinations of AR and mTORC1 inhibitors, an important therapeutic challenge [19,20].

Herein, our in vitro data show that Gal and the NGGAs are effective against both drug naïve and drug-resistant prostate cancer cell lines, suggesting a direct inhibitory effect on the neoplastic process. Our pharmacokinetics studies in mice established that the NGGAs exhibited improved pharmacokinetic profiles over Gal. Our antitumor efficacy data derived from the castration-resistant CWR22Rv1 tumor xenograft show the enhanced efficacy of the NGGAs over Gal, while ENZ is ineffective. Together, the in vitro and in vivo studies show that the drug naïve and drug-resistant PC cell lines are reliant on the AR/AR-V7 and Mnk-eIF4E signaling pathways for survival and proliferation. We propose that AR/AR-V7 and Mnk1/2 degraders could be effective in prostate cancer patients with upregulation of AR/AR-V7 and Mnk1/2 proteins, which currently represents a pressing therapeutic challenge [19,20,28,29,30,31].

## 2. Results

### 2.1. Unlike Enzalutamide, Galeterone and the Lead NGGAs Show Strong In Vitro Anti-Prostate Cancer Activities in Drug-Naïve and Drug-Resistant Prostate Cancer Cell Lines

**Figure 2:**
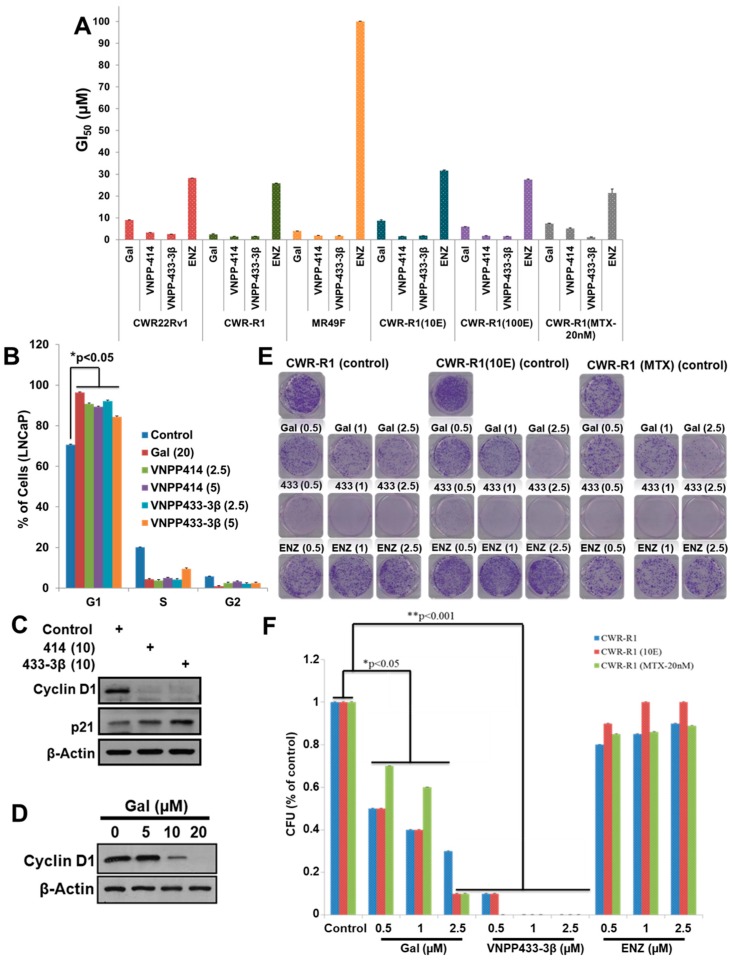
Gal and next generation galeterone analogs (NGGA) inhibit proliferation, colony formation and inhibit cell cycle progression of a variety of prostate cancer cell lines. (**A**) Comparative GI_50_ values of Gal, NGGAs, and enzalutamide (ENZ) in drug-naïve-/-resistant prostate cancer cells. (**B**) Gal and NGGAs induce G1 cell cycle arrest in LNCaP cells. (**C**) VNPP414 and VNPP433-3β deplete the cell cycle regulator, cyclin D1, and upregulate p21; “+” indicates specific treatments. (**D**) Gal cause dose-dependent depletion of cyclin D1. (**E**) Unlike ENZ, Gal and VNPP433-3β inhibit colony formation of drug-naïve/-resistant cells in vitro. 1000 cells/well (CWR-R1, CWR-R1 (10E), CWR-R1-MTX20nM), seeded in 6-well plates were treated with indicated concentrations of compounds for a period of 14 days. Media containing compounds were replaced every 3 days. Colonies were stained with 0.05% crystal violet. (**F**) Quantification of colony formation units (CFU) in drug-naive/resistant cells. Colony assays were repeated three times and colonies counted in four quadrants of the wells. Results are represented as averages with S.E.M. (* *p* < 0.05, ** *p* < 0.001). Note: the numbers in parentheses in Figure 2B,C,E are concentrations of the agents in µM.

The new NGGAs (VNPP414 and VNPP433-3β) were identified from in vitro anti-proliferative activities of our in-house compounds assessed in several PC cell lines, including LNCaP, C4-2B, and CWR22Rv1 (22Rv1) [8]. Because drug resistance remains a major clinical challenge in PC therapy [32,33,34,35,36,37], we compared the efficacies of Gal, VNPP414, and VNPP433-3β against several PC drugs-resistant cell lines, including enzalutamide (MDV-3100)-resistant (MR49F), mitoxantrone-resistant (CWR-R1(MTX), and docetaxel-resistant CWR-R1 (10E) PC cell lines. As shown in Figure 2A, Gal and the two lead NGGAs are more effective than ENZ against both the drug-naïve and drug-resistant cell lines. Notably, VNPP433-3β (GI_50_s = compounds concentrations required to inhibit cell growth by 50%, 1.12–2.54 µM) is 11-59 times more potent than MDV-3100 in the six different cell lines tested. We further demonstrated that the compounds induced G1 phase cell cycle arrest (Figure 2B) and caused marked depletion of cyclin D1 (an essential regulator of the G1–S transition) and strong up-regulation of p21 (cyclin-dependent kinase inhibitor) in LNCaP cells (Figure 2C,D)). It was recently reported that treatment of AR-expressing bladder cancer cell lines with enzalutamide induced up-regulation of tumor suppressor, p53, p21 and PTEN, and down-regulation of several oncogenic genes, such as c-myc, cyclin D1, and cyclin E [38]. VNPP414 and VNPP433-3β at 2.5 µM, each, are as effective as Gal at 20 µM (*p* < 0.05) (Figure 2B), indicating further the improved potencies (≥8-fold) of our new agents over Gal. We note that these cell-cycle studies were conducted in LNCaP cells. Inhibition of colony formations (clonogenicity assays) was also assessed by treating the parental CWR-R1 cells and two drug-resistant counterparts with Gal, VNPP433-3β, or ENZ. As shown in Figure 2E,F, the growth inhibitory trend seen in the antiproliferative assay was recapitulated, where VNPP433-3β was significantly more potent than Gal, while ENZ was ineffective at the concentrations tested.

### 2.2. Gal and VNPP433-3β Synergize with Docetaxel (DOC) and Enzalutamide (ENZ)

**Figure 3:**
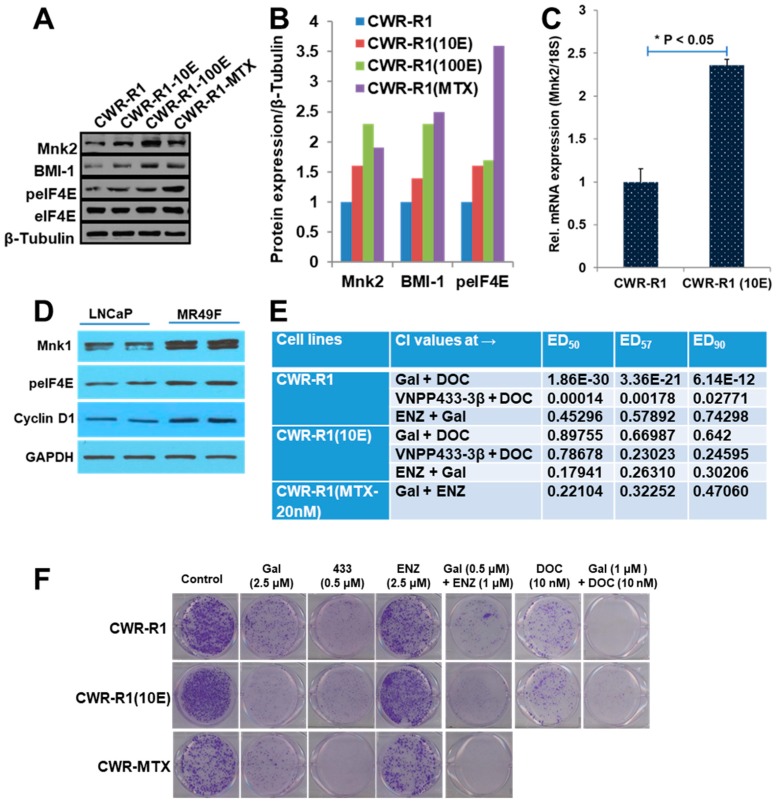
Characterization of drug-naïve/-resistant prostate cancer cells and effects of drug combinations with Gal or VNPP433-3β. (**A**) Western blot of protein expressions in CWR-R1, CWR-R1, (10E) and CWR-R1 (MTX-20nM) cells. (**B**) Densitometry of three representative experiments were averaged and plotted as bar charts. (**C**) Mnk2 mRNA expression in CWR-R1 and CWR-R1 (10E). (**D**) Western blot analysis of Mnk1, peIF4E, and cyclin D1 in LNCaP and ENZ-resistant MR49F cells. (**E**) Combination indices of agent interactions in prostate cancer cell lines. Cell viability assays were conducted for Gal, VNPP433-3β, ENZ, and docetaxel (DOC) individually and GI_50_ values calculated. Compounds were subsequently combined at their GI_50_ (constant ratio). Fractional effects of single agents and in combination were calculated and analyzed by Calcusyn software to compute the combination indices (CI) at ED_50_, ED_75_, and ED_90_. (CI < 1 -synergy, CI = 1 -additive and CI > 1 -antagonism) as previously described [9,10]. (**F**) Representative photographs of colonies formed in cells at 14^th^ day after initial treatment with the indicated compounds. Colonies were fixed with methanol and stained with crystal violet.

Based on reports that Mnk1/2-eIF4E and AR-V7 activation contributes to poor responses of PC to DOC or antiandrogens, and that Mnk1/2-eIF4E [39,40,41,42,43,44,45], or AR-V7 [46] inhibition induce chemo-sensitization in PC cells and our recent data that Gal/analogs and genetic targeting of Mnk1 and consequent BMI-1 depletion which has been implicated in DOC resistance in PC cells [47], we assessed if Gal and the NGGA would enhance the effects of DOC and ENZ in drug-sensitive CWR-R1 and drug-resistant CWR-R1 (10E) PC cells. Figure 3A,B clearly show up-regulation of drug-resistance biomarkers proteins, including, Mnk2, p-eIF4E, and BMI-1. Figure 3C shows significant elevation of Mnk2 mRNA levels in DOC-resistant CWR-R1 (10E) cells compared to the parental CWR-R1 cells, while Figure 3D shows significant upregulation of Mnk1 and cyclin D1 in the ENZ-resistant MR49F cells [48] compared to the parental LNCaP cells. We also show in Figure 3E that Gal and VNPP433-3β strongly synergize (CIs << 1) with DOC and ENZ in both cell lines. Furthermore, as shown in Figure 3F, Gal and VNPP433-3β markedly inhibited clonogenic ability of both cell lines and the combination treatment of Gal (0.5 µM) + ENZ (1 µM) or Gal (1 µM) + DOC (10 nM) completely suppressed colony formation, thus confirming strong synergic effects.

### 2.3. Gal and NGGAs Target AR/AR-V7 and Mnk1/2-eIF4E Signaling Pathways in Drug-Naïve and Drug-Resistant Prostate Cancer Cell Lines

**Figure 4:**
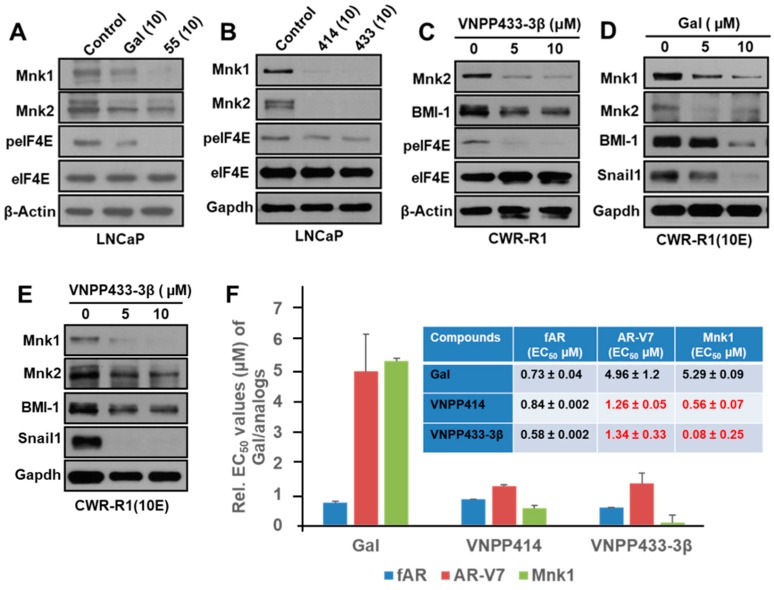
Impact of Gal and NGGAs on AR/AR-V7 and Mnk1/2-eIF4E signaling pathways. (**A**) Western blot showing the effects of Gal and VNPT55 (55) on Mnk1, Mnk2, and peIF4E. (**B**) Western blot showing the effects of VNPP414 (414) and VNPP433-3β (433)) on Mnk1, Mnk2, and peIF4E. (**C**) Western blot showing dose-dependent effect of VNPP433-3β (433) on Mnk2, BMI-1, and peIF4E in CWR-R1 cells. (**D**) Western blot showing dose-dependent effect of Gal on Mnk1, Mnk2, BMI-1, and Snail1 in drug-resistant CWR-R1(10E) cells. (**E**) Western blot showing dose-dependent effect of VNPP433-3β (433) on Mnk1, Mnk2, BMI-1, and Snail1 in drug-resistant CWR-R1(10E) cells. (**F**) EC_50_ values (for fAR, ARV-7 and Mnk1) for compounds determined from dose–response curves upon compound treatments (0–7.5 µM) of CWR22Rv1 cells for 72 h followed by western blot analysis of cells lysates. Note: the numbers in parentheses in Figure 4B are concentrations of the agents in µM.

**Figure 5:**
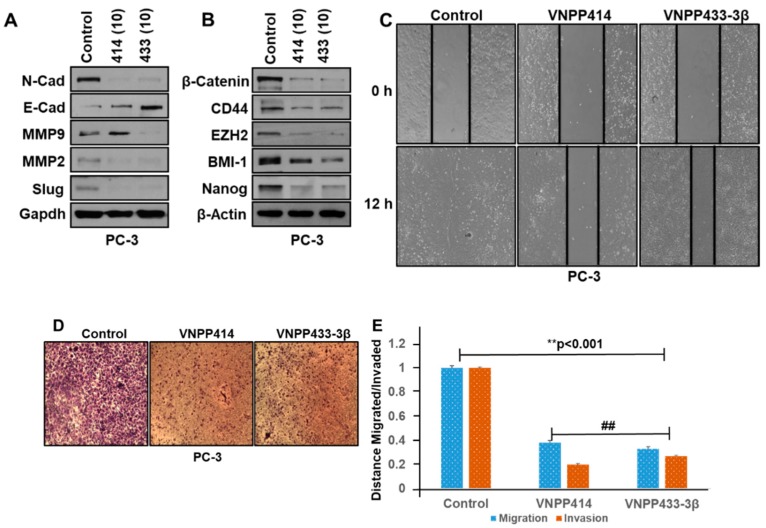
NGGAs modulate epithelial-to-mesenchymal transition (EMT) markers and inhibit cell migration and invasion. (**A**) VNPP414 (414) and VNPP433-3β (433) induce expression of E-cadherin and decreased expressions of N-cadherin, MMP2, MMP9, and Slug. (**B**) VNPP414 (414) and VNPP433-3β (433) decreased expressions of β-catenin, CD44, EZH2, BMI-1, and Nanog. (**C**) VNPP414 and VNPP433-3β inhibit PC cells migration. (**D**) VNPP414 and VNPP433-3β inhibit PC cells invasion. (**E**) Quantifications of inhibition of cell migration and invasion by VNPP414 and VNPP433-3β. Note: the numbers in parentheses in Figure 5A,B are concentrations of the agents in µM.

We had previously reported that VNPP414 and VNPP433-3β caused strong depletion of f-AR and AR-V7 in LNCaP and CWR22Rv1 cells. Here, we first explored the inhibitory effect of these two new analogs on Mnk1/2, eIF4E peIF4E and some downstream targets in four prostate cancer cell lines. Our results reveal that 24 h treatment of PC cells with Gal, VNPT55 [9], VNPP414, or VNPP433-3β significantly decreased the expression of Mnk1/2 with resultant suppression of p-eIF4E, without any significant effects on total eIF4E expression in LNCaP and CWR-R1 cells (Figure 4A–C). Figure 4D,E show that Gal and VNPP433-3β also caused marked dose-dependent suppression of Mnk1/2-eIF4E downstream target proteins, Snail 1 and BMI-1, in drug naïve- and DOC-resistant CWR-R1(10E) cells, respectively. Similar results were also obtained in PC-3 (Figure 5A,B), LNCaP, CWR22Rv1, and DU145 cells (Figure S3A–C).

We further determined the effects of lead NGGAs on f-AR, AR-V7 and Mnk1 protein levels in CWR22Rv1 cells. Following agent treatments (0–7.5 µM), the EC_50_ values were determined from dose–response curves. As shown in Figure 4F, the agents caused differential decreases in the levels of all three proteins. Of note is the finding that although there was no dramatic difference between the agents in terms of their effects on f-AR suppression, there were significant differences in their relative potencies against AR-V7 and Mnk1. Notably, VNPP414 and VNPP433-3β are 3.9 and 3.7 times and 9.4 and 66.0 times more effective in degrading AR-V7 and Mnk1 protein expressions, respectively. Given the known involvement of AR-V7 and/or Mnk1/2 upregulation in PC drug resistance, it is reasonable to suggest that the enhanced potencies of VNPP414 and VNPP433-3β against these oncogenic targets may contributing to their strong antiproliferative activities in the drug-naïve and drug-resistant cell lines reported in Figure 2A.

### 2.4. VNPP414 and VNPP433-3β Reverse EMT Activity, Deplete Stem Cell Like Factors and Inhibit Prostate Cancer Cells Migration and Invasion

Our recently published report strongly suggests that Gal and VNPT55 modulation of EMT and stem cell markers inhibits PC (PC-3, DU-145) cell migration and invasion [9]. Thus, we set out to examine whether the lead NGGAs, VNPP414 and VNPP433-3β, modulate the expression of EMT and the major putative stem cell markers. Treatments of PC-3 cells with these two agents caused strong and significant positive modulation of several EMT markers (decreases in N-cadherin, Slug, MMP2/9 and increase in E-cadherin) (Figure 5A) and significant suppression of the stem cell markers, β-catenin, CD44, EZH2, BMI-1, and Nanog (Figure 5B). We also demonstrated that Gal and the NGGAs decrease expressions of the EMT markers in docetaxel-resistant prostate cancer cells (see Figure 4D,E).

Thereafter, we demonstrated that our lead NGGAs also exert strong anti-migratory and anti-invasive activities in the PC-3 cell line which are noted for their high migratory and invasive potential [9]. As shown in Figure 5C,E, wound-healing assays clearly demonstrate that in control cells, 12 h after cell monolayers were wounded; cells filled the cleared areas. Treatments with VNPP414 or VNPP433-3β caused significant inhibition of PC-3 cells migration. As expected, these compounds also exerted strong anti-invasive activity against PC-3 cells (Figure 5D,E). Similar results (inhibition of cell migration) were also observed in DU-145 cells (Figure S4). We note that at the tested concentrations and duration (12 h) of assays, all cells in each treatment group were ≥ 95% viable, which suggests that the anti-migratory and anti-invasive activities of these agents were not due to cell cytotoxicity.

### 2.5. Pharmacokinetic Parameters of VNPP414 and VNPP433-3β in Mouse are Superior to Those of Galeterone

**Figure 6:**
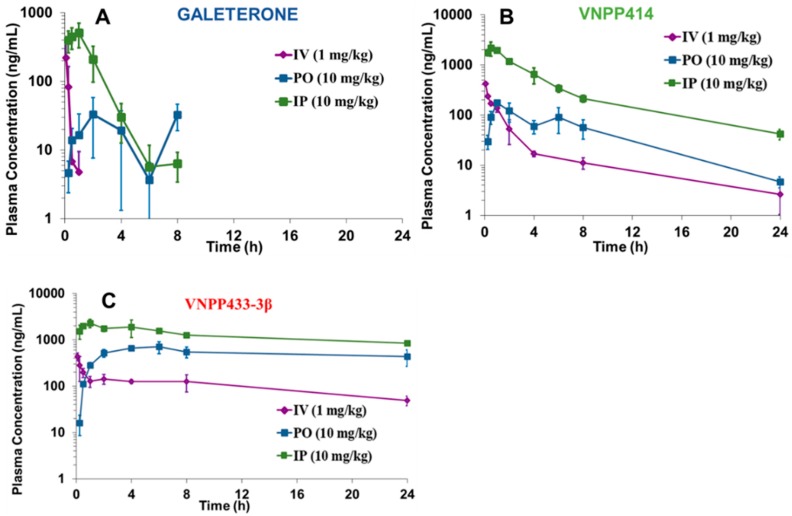
Plasma pharmacokinetic profiles of single IV, IP, and PO administrations of Gal, VNPP414 and VNPP433-3β to CD-1 mice. Values represent mean (±SD) concentrations from three mice.

Although we have previously reported the pharmacokinetics (PK) of Gal [49], we wanted to conduct a head-to-head (evaluated under the study same conditions) pharmacokinetic evaluation of Gal compared to out two lead NGGAs. Therefore, the PK of Gal, VNPP414, and VNPP433-3β after intravenous (IV), intraperitoneal (IP), and oral (PO) administration modes were investigated in male normal CD1 mice. Figure 6A–C depicts the plasma concentration versus time profiles, which clearly shows that unlike the NGGAs, Gal is rapidly cleared from the systemic circulation in mice (T_1/2_ = 0.17–1.26 h). The significant PK parameters are presented in Table 1. All three compounds exhibit >100% bioavailability (%F) following IP administration, which seems to be a characteristic for this class of compounds. In addition, the data clearly shows that VNPP433-3β has an excellent oral PK profile with good oral bioavailability (%F~50) in mice and achieves a C_max_ of 706.27 ng/mL (~2 µM) in the plasma, T_1/2_ of 31.2 h, AUC of 71,938.5 µM*h/L at 10 mg/kg oral dosing. Comparing their most important PK parameters (i.e., %F, C_max_, T_1/2_ and AUC), VNPP433-3β is 2.57-, 4.03-, 7.25-, and 30.0-folds, respectively, superior to VNPP414 and 2.69-, 21.5-, 24.75- and 285.34-folds, respectively, superior to Gal. It is highly likely that the enhanced PK profiles of our lead NGGAs contributed, in part, to their superior in vivo efficacies (vide infra).

### 2.6. Galeterone and the NGGAs Are More Effective Than Enzalutamide (ENZ) in Castration-Resistant Prostate Cancer CWR22Rv1 Xenograft Model

**Figure 7:**
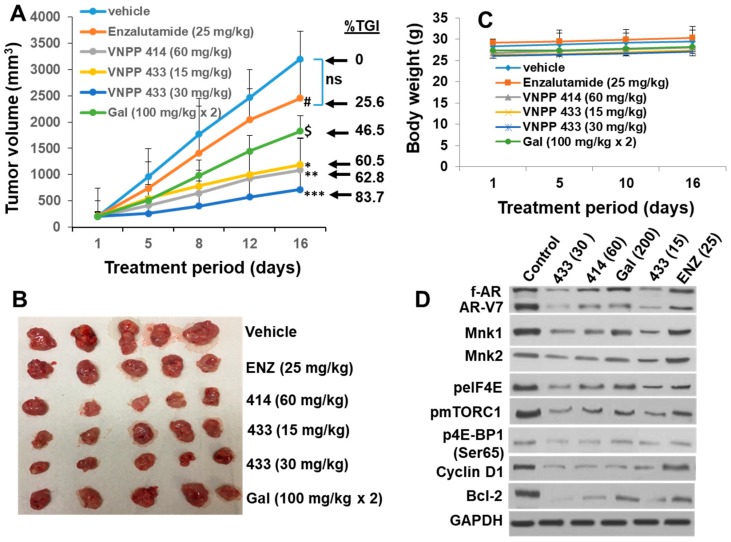
Effects of Gal, NGGAs and ENZ in an in vivo castration-resistant prostate cancer model. (**A**) Mice bearing CWR22Rv1 xenografts (5 mice per group) were treated with vehicle, Gal (100 mg/kg/twice daily), VNPP414 (60 mg/kg, once daily), VNPP433-3β (15 or 30 mg/kg, once daily) and ENZ (25 mg.kg, once daily) 5 days per week for 16 days. Tumor volumes were measured twice a week and the tumors were collected. Tumor growth inhibition (TGI) values are indicated at the right of each growth curve, and the error bars are the SEM. Results are represented as means ± SEM. ^#^ *p* = ns (not significant vs. vehicle), ^$^ *p* < 0.0272 vs. vehicle, * *p* < 0.0035 vs. vehicle, ** *p* < 0.0025 vs. vehicle and *** *p* < 0.0008 vs. vehicle. (**B**) Photographed tumors from each group. (**C**) Mean body weights of mice determined during the study. (**D**) Western blot analyses of tumor samples from each experimental group at day 16.

Following the in vitro studies and determination of the PK parameters, we then investigated the relative efficacy. Gal and the NGGAs in vivo using difficult-to-treat castration-resistant CWR22Rv1 xenografts. Given the enhanced IP and PO PK parameters of VNPP414 and VNPP433-3β over Gal, we decided to use lower doses of the new analogs compared to the previous Gal doses (200 mg/kg, IP/PO) [2]. Gal (200 mg/kg) served as positive control, while ENZ (25 mg/kg) administered PO was used as negative control [46,50]. Male NOD SCID mice bearing CWR22Rv1 tumors were treated with vehicle, ENZ, Gal, VNPP414, and VNPP433-3β for 16 days as described in the Methods and Materials Section. As shown in Figure 7A,B, CWR22Rv1 tumors were resistant to ENZ treatment, as there was no statistically significant difference in the tumor volumes between ENZ-treated and vehicle-treated control group. In contrast, Gal and the NGGAs caused statistically significant tumor growth inhibition (TGI). The decreasing order of potency (TGI) was as follows: VNPP433-3β (30 mg/kg; 83.7%, *p* < 0.0008 vs. vehicle) > VNPP414 (60 mg/kg; 62.8%, *p* < 0.0025 vs. vehicle) > VNPP433-3β (15 mg/kg; 60.5%, *p* < 0.0035 vs. vehicle) > Gal (200 mg/kg; 46.5%, *p* < 0.0272 vs. vehicle). It is notable that VNPP433-3β (at 7.5- and 15-fold lower molar doses) and VNPP414 (at 4-fold lower molar dose) are more efficacious than Gal. In general, no host toxicities were observed, as monitored by changes in animal body weight (Figure 7C). However, in the VNPP433-3β (30 mg/kg) treated group, one mouse died on day 16, with no preceding weight loss or other apparent cause.

To further validate the molecular mechanisms underlying the activities of Gal and the NGGAs, we evaluated the expression levels of f-AR, AR-V7, Mnk1, and Mnk2 and related downstream molecular targets. As shown in Figure 7D, VNPP433-3β was the most effective, causing dose-dependent decrease of f-AR, AR-V7, Mnk1, and Mnk2 and as expected decrease of p-eIF4E, the in vivo effector of Mnk1/2. Suppressions of these proteins correlated with down-regulation in the expressions of their downstream targets, including cyclin D1 and the antiapoptotic B-cell lymphoma 2 (Bcl-2). Treatments with VNPP433-3β also caused decrease of mTORC1 and one of its direct targets, p-4E-BP1. Gal (200 mg/kg) and VNPP414 (60 mg/kg) treatments also caused decrease in these proteins but to a lesser extent. Consistent with the lack of significant tumor growth inhibition, ENZ did not cause significant modulation of the molecular targets investigated.

## 3. Discussion

In this study, we have demonstrated that Gal and the NGGAs target the androgen receptor (including its splice variant AR-V7) and Mnk1/2-eIF4E (eIF4E phosphorylation at serine209) signaling pathways (dual AR/Mnk1/2 inhibitors) and that the NGGAs are more effective than Gal [8,9,11]. Because the androgen receptor is a negative regulator of eIF4E phosphorylation at serine 209 [19,20], our new class of compounds can effectively inhibit Mnk/eIF4E in addition to AR transcriptional activity with the potential to negate negative feedback to prevent PC progression. Additionally, because these two pathways are also implicated in de novo and acquired drug resistance, the NGGAs have the potential to thwart development of drug-resistance.

We show that Gal and the NGGAs exhibit profound anti-PC activities by inhibiting cell proliferation (of both drug-naïve and drug-resistant PC cells), colony formation, cell migration, invasion, and putative stem cell markers and reversed the expression of epithelial-to-mesenchymal transition (EMT), suggesting a direct inhibitory effect on the neoplastic process. In addition, we demonstrated that Gal and the NGGAs sensitized drug-resistant PC cells and in combination with docetaxel or enzalutamide synergistically inhibited drug-resistant PC cells, as evidenced by the low CI values. Mechanistically, these effects appear to be related to their abilities to inhibit the Mnk1/2-eIF4E axis and the downstream targets that are otherwise upregulated in these cell lines. The superior efficacy of the NGGAs compared to Gal may be attributed to their enhanced potencies against AR-V7 and Mnk1.

We also addressed the pharmacokinetic parameters of Gal and the NGGAs in mice in a head-to-head study. Our data clearly show that the pharmacokinetic parameters of VNPP414 and VNPP433-3β in mice are superior to those of Gal. Interestingly, the three compounds exhibit > 100% bioavailability (%F) following IP administration, suggesting complete bioavailability after IP administration. Although uncommon, there is literature precedence for several small molecules that exhibit absolute oral/IP bioavailability of >100% [51,52,53]. However, it is important to state here, that IP route of drug administration in humans is uncommon. With respect to the longer T_1/2_ values after IP or PO administrations compared to IV administration, a plausible reason may be that compounds from the depot site (i.e., site of IP or PO administration) may sustain drug levels in circulation if absorption is slow, potentially leading to long elimination half-lives. It is also possible that entero-hepatic recycling after oral administration may sustain blood levels of these molecules and prolong T_1/2_ [54,55]. Slow clearance due to slow metabolism of the compounds could also explain the observed phenomenon, which will be assessed in future tissue distribution studies.

The observed differences in the PK of Gal and its analogs may also be attributed to the differences in the chemical properties, including in vivo metabolic stabilities of the compounds. Unlike VNPP414 and VNPP433-3β with metabolically stable C-3 pyridyl methoxy and imidazole moieties, respectively, the rapid plasma clearance of Gal (with its Δ^5^, 3β-hydroxyl moiety) following oral administration is likely due to rapid metabolism by the two steroidogenic enzymes, 3βHSD and SRD5A, as previously reported by Alyamani and colleagues [56].

An important outcome of this study is that the effects of Gal and the NGGAs observed in vitro were recapitulated in vivo. Xenograft tumors derived from castration-resistant CWR22Rv1 cells which are resistant to ENZ treatment, were strongly inhibited by Gal and the NGGAs. Importantly, the NGGAs at significantly lower doses were more effective than Gal. The excellent half-life improvements of the NGGAs compared to the short half-life of Gal may also contribute to their dramatically lower effective doses, consistent with literature precedence [57]. Strikingly, the expressions of f-AR/AR-V7, Mnk1/2, peIF4E, and their associated target proteins, including cyclin D1 and anti-apoptotic Bcl-2, were strongly decreased in the Gal/NGGAs-treated tumors signifying inhibition of f-AR/AR-V7 and Mnk1/2-eIF4E signaling in the Gal/NGGAs-treated tumors as observed in vitro, thus validating their mechanisms of action. The results are similar to our recent report with novel retinamides that also target AR/AR-V7 and Mnk1/2-eIF4E [22]. The downregulation of p-mTORC1 and p4E-BP1 (a direct mTORC1 effector) is consistent with the recent findings that Mnk1/2 regulate mTORC1 signaling [58,59,60,61,62,63,64] and associate with mTORC1 directly [58,59].

Considering the findings of cross-resistance between taxanes (docetaxel and carbazitaxel) and the new antihormonal drugs abiraterone and ENZ [19], the lack of impaired efficacy of Gal and the NGGAs in the ENZ- and DOC-resistant cell lines suggest that Gal/NGGAs can be developed to treat men with PC that become resistant to all four drugs currently registered for use in mCRPC. Although Gal/NGGAs like the taxanes and the anti-hormonal agents interfere with AR-signaling, their lack of impaired efficacy in the resistant cell lines suggest that their ability to inhibit Mnk1/2-eIF4E signaling may represent a potential mechanism for the lack of cross-resistance. Finally, our study adds up to existing data on the oncogenic role of Mnk1/2-eIF4E signaling in prostate cancer [11,20,21,22,39,40,65]. Mnk1/2-eIF4E signaling is activated in the PC drugs-resistant cells, and inhibition of this pathway may restore ENZ and DOC functions in vitro and in vivo, leading to a better outcome. These data offer a precedent for the combination of dual AR/Mnk1/2 inhibitors with taxanes or ENZ and potentially other androgen deprivation therapies (ADTs).

## 4. Materials and Methods

### 4.1. Reagents, Compounds and Antibodies

Galeterone and analogs (VNPT55, VNPP414, and VNPP433-3β), and were all designed and synthesized in our laboratory as previously reported [3,8,49,66]. Enzalutamide was purchased from Sequoia Research Products, Pangbourne, RG8 7AP, UK and docetaxel and mitoxantrone was purchased from Cell Signaling. Compounds were either dissolved in dimethyl sulfoxide (DMSO) or 100% ethanol or kept at room temperature until they completely dissolved and stored at −20 °C.

Rabbit polyclonal antibodies against f-AR, AR-V7, β-actin, BMI-1, Gapdh, Mnk1/2, eIF4E, p-eIF4E, N-cadherin, E-cadherin, Snail, MMP-2/-9, secondary antibodies, anti-mouse and anti-rabbit HRP were purchased from cell signaling. was purchased from Sigma Aldrich. Oct-4 and Nanog were kind donations from Dr. Raju Khatri of the Department of Biochemistry and Molecular Biology, University of Maryland, Baltimore. EZH2 mouse monoclonal antibody was from Dr. Yun Qiu, Department of Pharmacology, University of Maryland, Baltimore, MD, USA.

### 4.2. Cell Culture

The human prostate cancer cells lines, LNCaP, PC-3, DU145, CWR22Rv1 were purchased from American Type Culture Collection (ATCC, Manassas, VA, USA, and were maintained in RPMI supplemented with 10% feta bovine serum (FBS) and 1% penicillin-streptomycin. CWR-R1, CWR-R1 (10E), CWR-R1 (100E), and CWR-R1 (MTX-20nM) prostate cancer cells were obtained from Dr. Yun Qiu of the Department of Pharmacology, University of Maryland, Baltimore. CWR-R1 cells were made resistant to docetaxel at 10 nM (CWR-R1 (10E)), 100 nM (CWR-R1 (100E)) and to Mitoxantrone at 20 nM (CWR-R1 (MTX-20nM)) [67,68]. These cells were maintained in regular phenol red RPM1 supplemented with 10% heat inactivated FBS and 1% penicillin-streptomycin antibiotics. The drug-resistant cells were maintained in media supplemented with the respective compounds at the resistant concentrations. MR49F, Enzalutamide resistant LNCaP cells (a generous gift of Dr. Amina Zoubeidi, The Vancouver Prostate Centre) were maintained in RPMI 1640 supplemented with 10% fetal bovine serum, 1% penicillin/streptomycin and 10 µM Enzalutamide.

### 4.3. Western Blot Analysis

Cells were lysed with radioimmunoprecipitation assay (RIPA) buffer (Sigma, St. Louis, MO, USA), supplemented with 1X protease inhibitors (Roche, Indianapolis, IN, USA), phosphatase inhibitors (Thermo Scientific, Waltham, MA, USA), 1 mmol/L EDTA and 1 mmol/L PMSF (Sigma). Western blot analyses were performed as in Kwegyir-Afful et al., 2015 [2].

### 4.4. Cell Viability Assays MTT (3-(4, 5-dimethylthiazol-2-yl)-2, 5-diphenyltetrazolium Bromide, Colorimetric Assay)

MTT assays were performed as described in our previous publications [3]. Briefly, 2500 cells were seeded in 96 well plates overnight. Cells were then treated with serially diluted compounds in culture media for 7 days. Data was analyzed with GraphPad prism 4 software.

### 4.5. Combination Studies to Assess Synergy, Additivity or Antagonism

To analyze interactions between Gal/analogs with docetaxel or ENZ, MTT cell viability assays were performed for the indicated compounds with the respective cell lines. The GI_50_ values were calculated using graphpad prism. Two different compounds were combined at a constant ratio of their GI_50_ values. The fraction of cells affected after treatment period was analyzed as described previously [69]. The calcusyn software was used to determine the combination index (CI), where CI < 1 indicate synergy, CI = 1 indicates additive and CI >1 indicates antagonism.

### 4.6. RNA Isolation and Real-Time Polymerase Chain Reaction Analysis

Cells were seeded in 6-well plates at 0.3 × 10^6^ cells per well. Ribonucleic acid (RNA) was isolated with the Qiagen RNeasy reagents following manufacturer’s protocol. Eighteen hundred (1800) ng of RNA were reverse transcribed into cDNA using high capacity cDNA reverse conversion kit (Life Technologies, Carlsbad, CA, USA). Relative mRNA levels of Mnk2 and were quantified with the comparative ΔΔC_t_ using 18S as internal control.

### 4.7. Cell Motility and Invasion Assay

Trans-well Boyden chamber, 8-µm pore size, (BD Biosciences, San Jose, CA, USA) pre-coated with basement membrane extract (BME) and maintained room temperature for 2 h. In invasion assays, 1 × 10^4^ cells/well, were seeded in the top chamber in serum-free media with or without indicated concentrations of compounds. The bottom chamber was filled with 600 µl of regular media with 10% fetal bovine serum. Experimental set-up was placed in a 37 °C incubator for 24 h. Cells at the top chamber were scraped off with cotton swabs and migrated cells at the bottom of the inserts were fixed with ice cold methanol and stained in 0.05% crystal violet. In motility experiments inserts used were not coated with.

### 4.8. Colony Formation Assay

0.1 × 10^3^ cells per well were seeded in 6-well plates and allowed to attach overnight (16 h), cells were treated with compounds in regular media (RPMI with 10% FBS) at the indicated concentrations and replaced every 3rd day for 14 days or until visible colonies could be counted. Colonies were washed and stained with 0.05% crystal violet for 30 min. Colonies were scanned and quantified (colonies counted in four quadrants of each well) with ImageJ colony counter. Results are represented as the mean of triplicates.

### 4.9. Animal Study Approval

The pharmacokinetics study was conducted at GVK BIO., Hyderabad, India, in accordance with Study Protocol No.: 137-17-DMPK, 138-17-DMPK and 452-17-DMPK (14 December 2016). This study was performed after approval from the Institutional Animal Ethics Committee (IAEC) (proposal no.: B-011) in accordance with the requirement of Committee for Control and Supervision of Experiments on Animals (CPCSEA), India (1125/PO/Rc/S/07/CPCSEA).

The antitumor efficacy studies in mice were performed according to protocol #1217012 reviewed and approved by the Institutional Animal Care and Use Committee (IACUC) at the University of Maryland School of Medicine, Baltimore, MD, USA, (IACUC No. 1217012, 12 January 2018).

#### 4.9.1. Pharmacokinetic Studies

These studies were conducted in male CD-1 mice (7–8 weeks old, weighing about 27–33 g) were obtained from Hylasco Bio-Technology Pvt. Ltd. (A Charles River Technology Licensee, Turkapally, India). The details are presented in the Supplementary Materials.

#### 4.9.2. In Vivo Anti-Tumor Studies in CWR22Rv1 CRPC Xenograft Model.

Male NOD SCID mice 5–6 weeks of age were obtained from University of Maryland School of Medicine Veterinary Resources (Baltimore, MD, USA. Mice were housed under complete aseptic conditions, fed autoclaved pellets and sterile water ad libitum. Following a week of acclimatization, approximately 5 × 10^6^ CWR22Rv1 cells were inoculated one flank f mice. Tumor-bearing mice (tumor volume around ~100 mm^3^) were randomized into 6 groups (5 mice in each group; compounds formulated in vehicle) and treated as follows: (i) vehicle control (40% β-cyclodextrin in saline, PO.), (ii) ENZ (25 mg/kg, PO, once daily), (iii) Gal (100mg/kg, PO, twice daily), (iv) VNPP414 (60 mg/kg, PO, once daily), (v) VNPP433-3β (15 mg/kg, PO, once daily), and (vi) VNPP433-3β (30 mg/kg, PO, once daily) for 5 days/week. Tumors were measured twice weekly with calipers and tumor volume was calculated by the formula: length × width^2^ × 0.5 (mm^3^). Animals were also weighed weekly and monitored for general health status and signs of possible toxicity due to treatments. Mice were sacrificed after the indicated periods of treatment and tumors excised. Tumors were divided and either flash frozen in liquid nitrogen or placed in 10% buffered formalin for western blot analysis.

### 4.10. Statistical Analysis

Experiments were performed in triplicates (at least) and presented as means with standard error of the means (S.E.M) where applicable. T-test significance was performed with a boundary of *p* < 0.05.

## 5. Conclusions

The results presented herein demonstrate that the NGGAs, VNPP414 and VNPP433-3β, are promising anti-prostate cancer molecules and are more effective than their parent molecule, Gal and the FDA-approved anti-AR drug, enzalutamide. The NGGAs were shown to possess enhanced pharmacokinetics parameters and inhibitory potencies against AR/AR-V7 and Mnk1/2-eIF4E signaling pathways. The in vitro results, especially their activities against drug-resistant prostate cancer cell lines were successfully translated in vivo in a CWR22Rv1 cell-derived xenograft model following oral administration of the agents. Collectively, these results classify VNPP414 and VNPP-433-3β as valuable preclinical lead candidates for further evaluation in prostate cancer clinical phase 1 studies.

## Figures and Tables

**Table 1:** Pharmacokinetic parameters of Galeterone, VNPP414 and VNPP433-3β after intravenous (IV), intraperitoneal (IP), or oral (PO) administration in male CD-1 mice.

Compounds	Dosing	AUC (0–∞) (ng.h/mL)	C_max_ (ng/mL)	T_1/2_ (h)	F (%)
**Galeterone**	IV (1 mg/kg)	57.90	-	0.17	-
	IP (10 mg/kg)	111.29	506.59	1.24	168.81
	PO (10 mg/kg)	969.07	32.8	1.26	18.44
**VNPP414**	IV (1 mg/kg)	547.43	-	7.48	-
	IP (10 mg/kg)	8601.34	2174.37	6.25	157.12
	PO (10 mg/kg)	1030.00	175.40	4.30	19.35
**VNPP433-3β**	IV (1 mg/kg)	3469.23	-	14.26	-
	IP (10 mg/kg)	57290.45	2294.19	22.38	123.20
	PO (10 mg/kg)	31755.01	706.27	31.19	49.45

AUC(0–∞): area under the concentration-time curve from the time of dosing extrapolated to infinity; C_max_: maximum plasma concentration; T_1/2_: elimination half-life and F (%): absolute bioavailability.

## Supplementary Materials

The following are available online at https://www.mdpi.com/2072-6694/11/11/1637/s1, Table S1: Optimized mass spectroscopic conditions; Table S2: Calculated concentrations and % accuracy for galeterone calibration standards prepared in mice plasma (*n* = 3); Table S3: Calculated concentrations and % accuracy for VNPP414 calibration standards prepared in mice plasma (*n* = 3); Table S4: Calculated concentrations and % accuracy for VNPP433-3β calibration standards prepared in mice plasma (*n* = 3); Figure S1: Gal, VNPP414 or VNPP433-3β calibration curves in plasma; Figure S2: LC-MS/MS chromatograms of plasma samples obtained after Gal, VNPP414 or VNPP433-3β administration from IV (0.038 h), PO (0.25 h) or IP (0.25 h) to CD-1 mice; Figure S3: NGGAs modulate EMT markers; Figure S4: VNPP414 and VNPP433-3β inhibit DU-145 cells migration.
