# Supplementary material for: Galeterone and The Next Generation Galeterone Analogs, VNPP414 and VNPP433-3β Exert Potent Therapeutic Effects in Castration-/Drug-Resistant Prostate Cancer Preclinical Models In Vitro and In Vivo
Source: Cancers (Basel). 2019 Oct 24;11(11):1637. doi: 10.3390/cancers11111637 (PMC6895912; doi:10.3390/cancers11111637)
Supplement: Supplementary file 1 [file cancers-11-01637-s001.pdf]

cancers

MDPI

Supplementary material

Galeterone and Next Generation Galeterone Analogs, VNPP414 and VNPP433-3β Exert Potent Therapeutic Effects in Castration-/Drug-Resistant Prostate Cancer Preclinical Models In Vitro and In Vivo

1. Materials and Methods for Pharmacokinetics studies

The study was conducted at GVK BIO., Hyderabad, India, in accordance with Study Protocol No.: 137-17-DMPK, 138-17-DMPK and 452-17-DMPK. This study was performed after approval from the Institutional Animal Ethics Committee (IAEC) in accordance with the requirement of Committee for Control and Supervision of Experiments on Animals (CPCSEA), India.

1.1. Steroidal Compounds, Reagents, Solvents and Materials

Galeterone (Gal), VNPP414 andVNPP433-3β were designed and synthesized in our laboratory following procedures reported previously [20-22]. VNPP433-3β was used as internal standard for Gal studies, while Gal was used as internal standard for VNPP414 andVNPP433-3β studies. HPLC grade acetonitrile, methanol, water, ammonium acetate and formic acid (90% pure) were purchased from Merck specialties Pvt Ltd (Mumbai, India).

All mass spectrometric estimations were performed on a sciex API-4500 triple quadrupole instrument with turboionspray (AB Sciex, Toronto, Canada). The HPLC system consisted two of LC 20 AD pumps, a SIL-20 AC-HT autosampler, a Rack Changer II and CDM 20A controller (Shimadzu, Kyoto, Japan). The stationary phase for Gal analogs was XBridge C18 with 3.5 μm particle diameter (Waters, Ireland) while for Gal the stationary phase was Kinetex, C18 with 4 μm particle diameter (Waters, Ireland). The column dimensions were 50 × 4.6 mm.

1.2. Preparation of Calibration Standards and Quality Control Samples

Master stock solutions of Gal/analogs (analytes/IS; purity 99%) (1 mg/mL) were prepared in dimethyl sulfoxide (DMSO). Master stock solutions were stored at 4 ^{0}C when not in use. In general, working standard solutions were prepared at 25-fold higher concentrations than plasma calibration standards and quality control samples. A total of nine calibration standards and three quality control (QC) samples were prepared. Plasma calibration standards (1.03, 2.06, 10.31, 51.54, 206.18, 515.44, 824.71, 926.64, 1029.60 ng/mL) and QC samples (4.53, 522.72, 871.20 ng/mL) of Gal was prepared by spiking 2.0 μL of calibration curve standards into 48 μL of blank matrix followed by precipitation with 200 μL of acetonitrile containing internal standard (VNPP433-3β) at 200 ng/mL, vortexed for 5 min at 850 rpm, centrifuged at 4000 rpm for 5 min at 4 °C. 110 μL of supernatant was separated and diluted with 110 μL of methanol:water (1:1, v/v). For VNPP414 and VNPP433-3β assays, the plasma calibration standards (1.00, 2.00, 10.01, 50.06, 200.26, 500.64, 801.03, 900.04, 1000.04 ng/mL) and QC samples (4.53, 522.72, 871.20 ng/mL) were utilized. Gal (200 ng/mL) was used as the internal standard.

1.3. Sample Preparation

A 50 μL aliquot of plasma (blank control plasma, plasma samples from mice dosed with Gal/analogs, blank plasma spiked with calibration standards and QC samples) was taken and precipitated with 200 μL of acetonitrile containing internal standard at 200 ng/mL. Samples were then vortexed for 5 min at 850 rpm, centrifuged at 4000 rpm for 5 min at 4 °C. 110 μL of supernatant was separated and diluted with 110 μL of methanol:water (1:1,v/v) , and 5 and 10 μL aliquots for Gal and analogs assays, respectively, were injected for LC-MS/MS analysis.

1.4. LC-MS/MS Analysis

A generic gradient LC method with a short run times of 3.5 min for Gal and 4.5 min for VNPP414 and VNPP433-3β were developed for the analysis of Gal/analogs in plasma samples. The mobile phase flow rate was 1.0 mL/min with a split ratio of 1:1 to the ionization source. The mobile phase for Gal consisted of 10 mM ammonium acetate in water as aqueous component and 100% methanol as organic modifier. For the Gal analogs, the mobile phase consisted of 0.01% formic acid in water and 100% acetonitrile. The column and autosampler were maintained at 40°C and 15°C, respectively for all assays. The turbo ion spray source was operated with typical settings as follows: ionization mode, positive; curtain gas, 30 psi; nebulizer gas (GS1), 55 psi; heater gas (GS2), 50 (for Gal analyte) and 60 (for Gal analogs analytes) psi; nebulizer gas 55 psi; temperature, 550 °C. A summary of the ion transitions, collision energies and retention times for both the analytes (Gal, VNPP414 and VNPP433-3β) and the internal standards (IS, VNPP433-3β and Gal) are presented in Table S1. Peak areas for all components were automatically integrated using Analyst software version 1.5.

Table S1. Optimized mass spectroscopic conditions. *IS: internal standard; **Gal had two daughter ions (m/z: 119.20 and 195.20) that were used for multiple reaction monitoring (MRM) in LC-MS/MS runs. Because we observed a poor shape peak of Gal with fragment of m/z 195.20 at lower levels (LLOQ: 1 ng/mL) in plasma samples, we used fragment m/z 119.20 for the PK study. We used fragment of m/z 195.20 when Gal was used as IS where the concentration was ~200 ng/mL. This fragment was stable throughout the study.

Analytes	Parent ions (m/z)	Daughter ions (m/z)**	Collision energy (V)	Declustering potentials (V)
Gal	389.10	119.20	62	147
VNPP433-3β (IS)*	439.20	371.30	18	82
VNPP414	480.20	195.20	82	110
Gal (IS)*	389.20	195.20	66	100
VNPP433-3β	439.30	371.50	30	110
Gal (IS)*	389.20	195.20	66	100

1.5. Pharmacokinetics of Gal, VNPP414 and VNPP433-3β in CD-1 Mice

Male CD-1 mice (7-8 weeks old, weighing about 27-33 g) were obtained from Hylasco Bio-Technology Pvt. Ltd (A Charles River Technology Licensee in India) were maintained in a controlled environment of about 25 °C, 50% relative humidity and a 12-h light/12-h dark cycle. The compounds were formulated in either 10% DMSO in 10% solutol in phosphate-buffered saline (PBS) for single intravenous doses (1 mg/kg) or in 40% β-cyclodextrin in saline for single intraperitoneal or oral doses (10 mg/kg). 10 ml/kg dose volume used for administrating the formulation for all the three routes (IV, IP and PO). The IV administration was performed through tail vein of mice as bolus. For IP route, the compound was dosed in intraperitoneal cavity using a 27-gauze needle. PO administration was performed through gastric gavage tube (aka feeding tube). Serial blood samples were collected from 3 mice per time-point (staggered bleeding approach was used for mice where one mice was bled maximum for three time within 24h) into vacutainers containing lithium heparin (anticoagulant) at 0.083, 0.25, 0.50, 1, 2, 4, 8 and 24 h post dose after intravenous administration and 0.25, 0.50, 1, 2, 4, 8 and 24 h post dose after intraperitoneal or oral administration. In total, 81 mice were used for this study. At each time point 200 μL of blood was collected into vacutainers. Venous blood Samples were collected using retro orbital puncture method. Plasma was isolated by centrifugation at 10000 rpm for 10 min and stored frozen at -80°C until assay. Plasma pharmacokinetic parameters were calculated using the non-compartmental analysis tool of Phoenix software (Version 7, Pharsight) and were determined from individual animals in each group. The area under the plasma concentration-time curve ($AUC_{0\text{-}t}$ and AUC_{inf}), elimination half-life ($T_{1/2}$), clearance (CL), volume of distribution (V_d) and mean residence time (MRT) were calculated from intravenous group. The peak plasma concentration (C_{max}), time to achieve peak plasma concentration (T_{max}), $AUC_{0\text{-}t}$ and AUC_{inf}, were

calculated for the IP and PO groups. Absolute oral bioavailability (%F) were calculated from the PO group. %F was calculated using the equation: $F=((AUC_{ip/po}/AUC_{iv})*(Dose_{iv}/Dose_{ip/po}))*100$. This equation is appropriate for non-compartmental methods although there are some limitations for drugs with non-linear PK. It does not assume any particular mechanism of elimination. In addition, the formula used for calculating %F also holds true for single dose administration.

1.6. LC-MS/MS Analysis of Gal and Analogs

Gal, VNPP414 and VNPP433-3β were well resolved from their respective internal standards by reverse-phase HPLC methods used. Under the optimized LC-MS/MS conditions, no interference at the retention times of Gal (2.07 min) and its internal standard VNPP433-3β (1.91 min). For VNPP414 and VNPP433-3β, the retention times were 2.44 and 1.91 min, respectively compared to internal standard Gal (2.07 min). The calibration curves derived for each compound were linear and reproducible and the inter- and intra-assay variabilities were less than 20% (Supplementary Tables S2- S4 and Supplementary Figure S1A–C). The linear regression equations for Gal, VNPP414 and VNPP433-3β from the lowest to the highest concentration's ranges were: y = 0.00037x+0.000572, r = 0.9963; y = 0.000474x + 0.000511, r = 0.9966 and y =0.000474x + 0.000511, r = 0.9966, respectively. The lowest limits of quantification (LLOQ) for Gal, VNPP414 and VNPP433-3β in mouse plasma were 1.03, 1.00 and 1.00 ng/mL, respectively. The LC-MS/MS assays were fully validated and used to monitor the concentrations of each compound in mouse plasma.

Table S2. Calculated concentrations and % accuracy for galeterone calibration standards prepared in mice plasma (n = 3). (*): Outlier: Back Calculated concentration deviated by more than 20%; STD = calibration curve standard; HQC = higher quality control; LLOQ = lower limit of quantification; ULOQ = upper limit of quantification.

Standard	Concentration [ng/mL]		% Accuracy
	Nominal	Calculated	
STD 1 (LLOQ)	1.03	1.13	109.13
STD 2	2.06	1.69	81.87
STD 3	10.31	10.27	99.64
STD 4	51.54	50.47	97.91
STD 5	206.18	205.92	99.87
STD 6	515.44	498.29	96.67
STD 7	824.71	869.73	105.46
STD 8	926.64	956.16	103.19
STD 9 (ULOQ)	1029.60	1094.01	106.26
Correlation (r)	0.9963		
Regression equation	Y =0.00037x+0.000572		
Regression Fit	Linear with 1/x2 weighting factor		

Quality Control Set		Concentration (ng/mL)		% Accuracy
		Nominal	Calculated	
HQC	HQC-1	871.20	994.23	114.12
	HQC-2		958.72	110.05
MQC	MQC-1	522.72	560.32	107.19
	MQC-2		589.16	112.71
LQC	LQC-1	4.53	4.51	99.50
	LQC-2		4.43	97.71

Table S3. Calculated concentrations and % accuracy for VNPP414 calibration standards prepared in mice plasma (n = 3). (*): Out of specification; STD = calibration curve standard; HQC = higher quality control; LLOQ = lower limit of quantification; ULOQ = upper limit of quantification.

Standard	Concentration [ng/mL]		% Accuracy
	Nominal	Calculated	
STD 1 (LLOQ)	1.00	0.98	97.69
STD 2	2.00	2.09	104.30
STD 3	10.01	10.08	100.70
STD 4	50.06	51.41	102.70
STD 5	200.26	223.65	111.68
STD 6	500.64	516.03	103.07
STD 7	801.03	673.62	84.09
STD 8	900.04	848.17	94.24
STD 9 (ULOQ)	1000.04	1015.38	101.53
Correlation (r)	0.9966		
Regression equation	Y =0.000474x+0.000511		
Regression Fit	Linear with $1/x^2$ weighting factor		

Quality Control Set		Concentration (ng/mL)		% Accuracy
		Nominal	Calculated	
**HQC**	HQC-1	880.03	865.66	98.37
	HQC-2		819.6	93.13
**MQC**	MQC-1	528.02	537.33	101.76
	MQC-2		511.81	96.93
**LQC**	LQC-1	4.93	6.16	124.91*
	LQC-2		5.49	111.32

Table S4. Calculated concentrations and % accuracy for VNPP433-3β calibration standards prepared in mice plasma (n = 3). (*): Outlier: Back Calculated concentration deviated by more than 20%; (**) Out of specification; STD = calibration curve standard; HQC = higher quality control; LLOQ = lower limit of quantification; ULOQ = upper limit of quantification.

Standard	Concentration [ng/mL]		% Accuracy
	Nominal	Calculated	
STD 1 (LLOQ)	1.00	1.10	109.91
STD 2	2.00	1.63	81.27
STD 3	10.01	9.34	93.31
STD 4	50.06	52.45	104.78
STD 5	200.26	198.23	98.99
STD 6	500.64	601.52*	120.15*
STD 7	801.03	783.67	97.83
STD 8	900.04	865.47	96.16
STD 9 (ULOQ)	1000.04	976.04	97.60
Correlation (r)	0.9966		
Regression equation	Y =0.000474x+0.000511		
Regression Fit	Linear with $1/x^2$ weighting factor		

Quality Control Set		Concentration (ng/mL)		% Accuracy
		Nominal	Calculated	
HQC	HQC-1	880.03	603.00	68.52**
	HQC-2		730.90	83.05
MQC	MQC-1	528.02	478.90	90.70
	MQC-2		497.04	94.13
LQC	LQC-1	4.93	4.23	85.72
	LQC-2		4.07	82.46

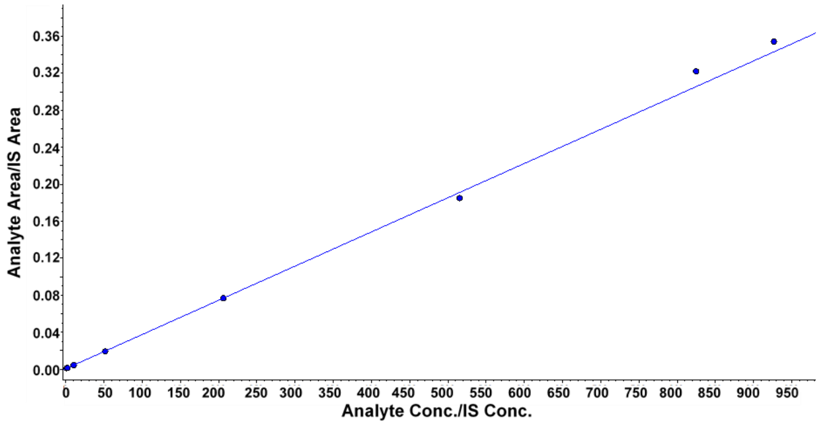

Figure S1A. Galeterone calibration curve in plasma.

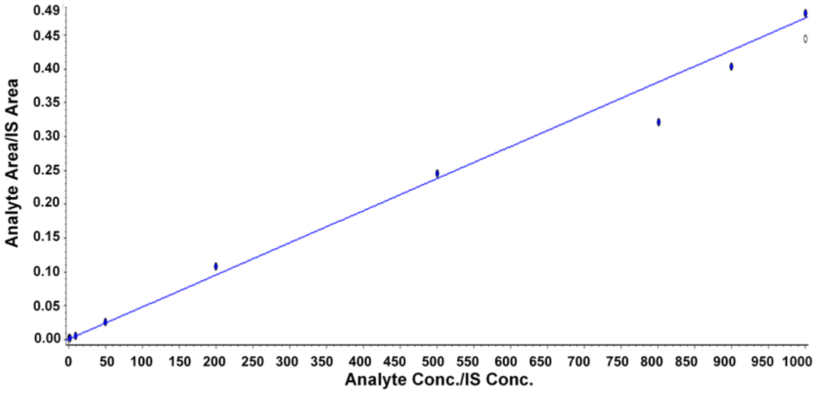

Figure S1B. VNPP414 calibration curve in plasma.

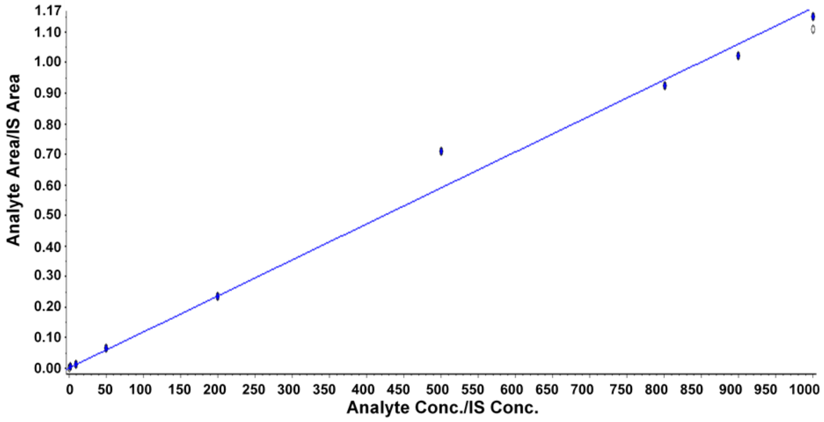

Figure S1C. VNPP433-3β calibration curve in plasma.

Figure S1. Gal, VNPP414 or VNPP433-3β calibration curves in plasma.

1.7. Pharmacokinetics of Gal and Analogs

The concentrations of Gal, VNPP414 and VNPP433-3β were successfully determined following administration of single doses the three compounds to CD-1 male mice (n = 3 animals per time point) through intravenous (IV, 1.0 mg/kg, each), intraperitoneal (IP, 10 mg/kg each) or oral (PO, 10 mg/kg each) administrations. Representative chromatograms of Gal from IV (0.083 h), PO (0.25 h) or IP (0.25 h); VNPP414 from IV (0.083 h), PO (0.25 h) or IP (0.25 h) or VNPP433-3β from IV (0.083 h), PO (0.25 h) or IP (0.25 h) are shown in Figure S2A–C, respectively.

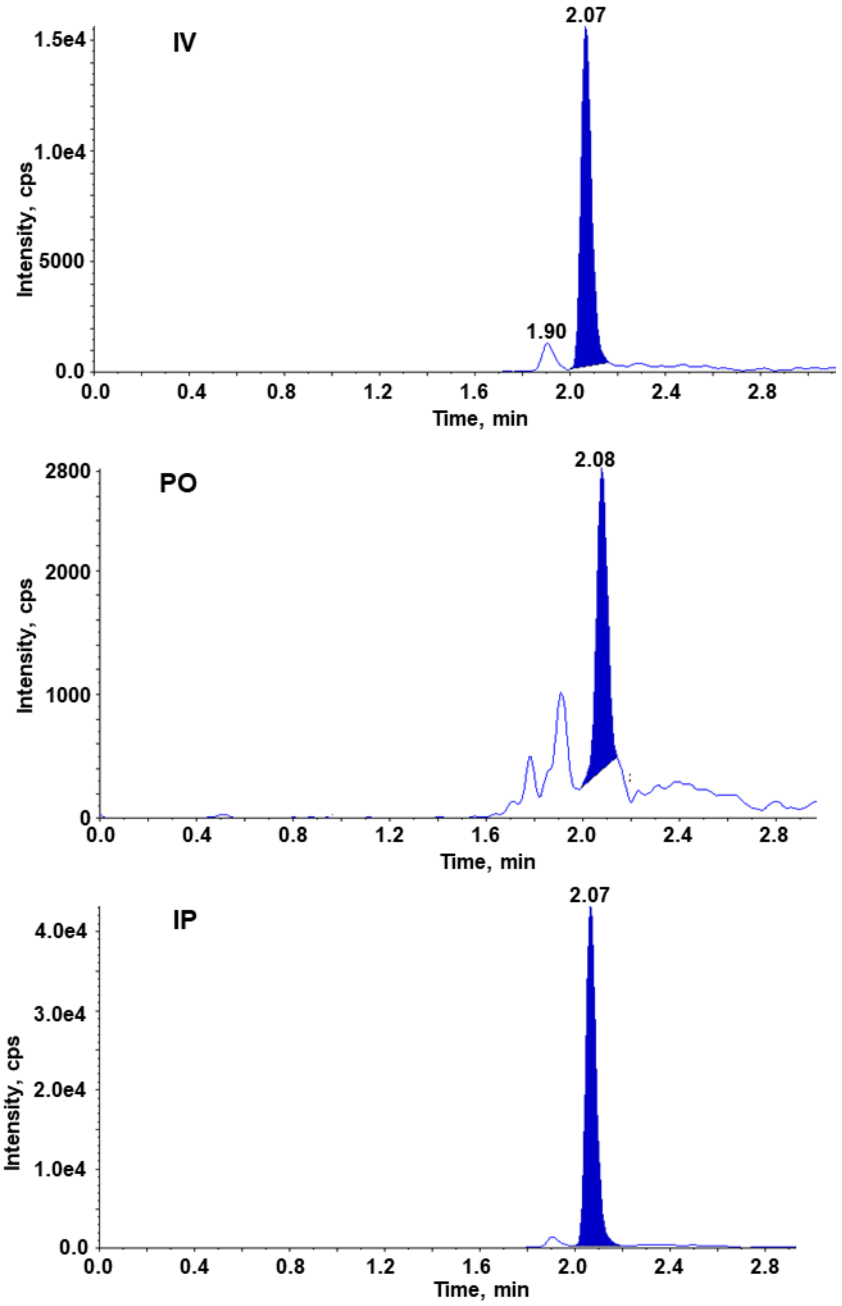

Figure S2A. LC-MS/MS chromatograms of plasma samples obtained after Gal administration from IV (0.038 h), PO (0.25 h) or IP (0.25 h) to CD-1 mice.

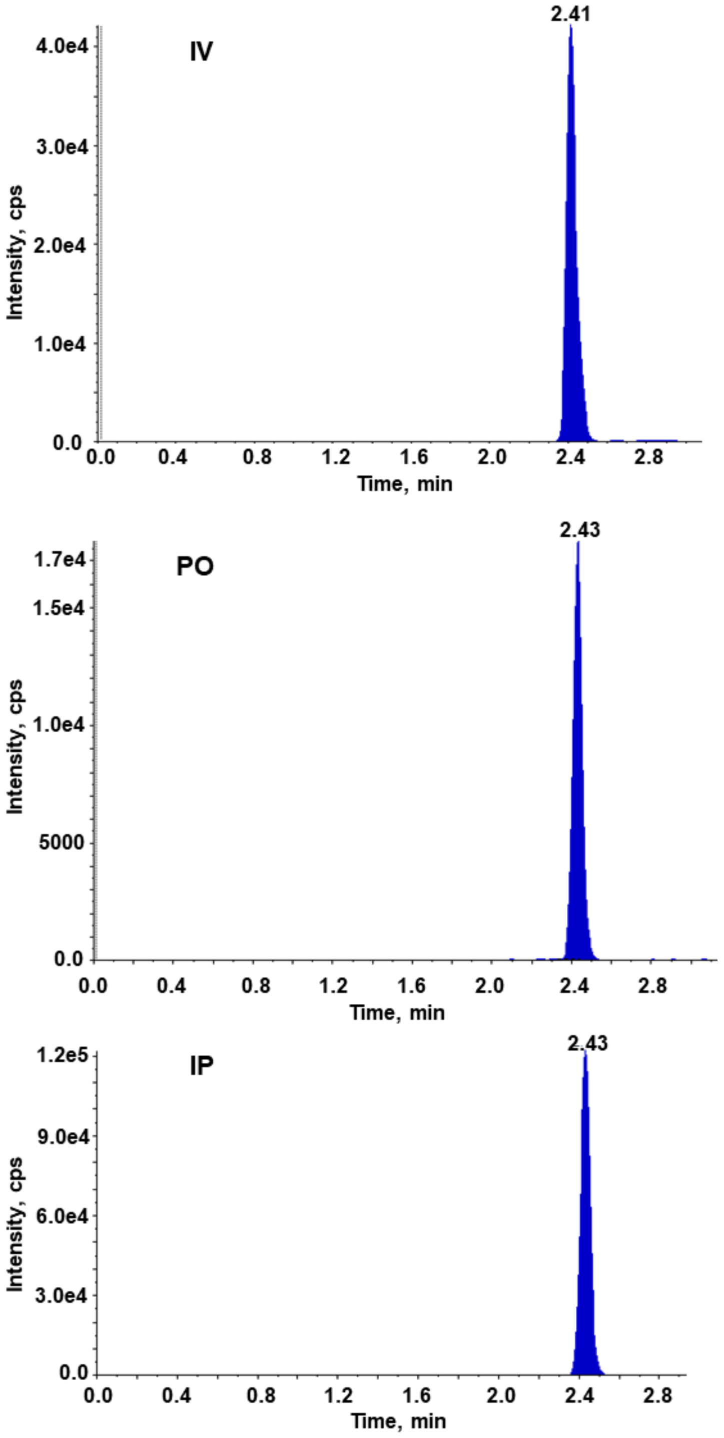

Figure S2B. LC-MS/MS chromatograms of plasma samples obtained after VNPP414 administration from IV (0.083 h), PO (0.25 h) or IP (0.25 h) to CD-1 mice.

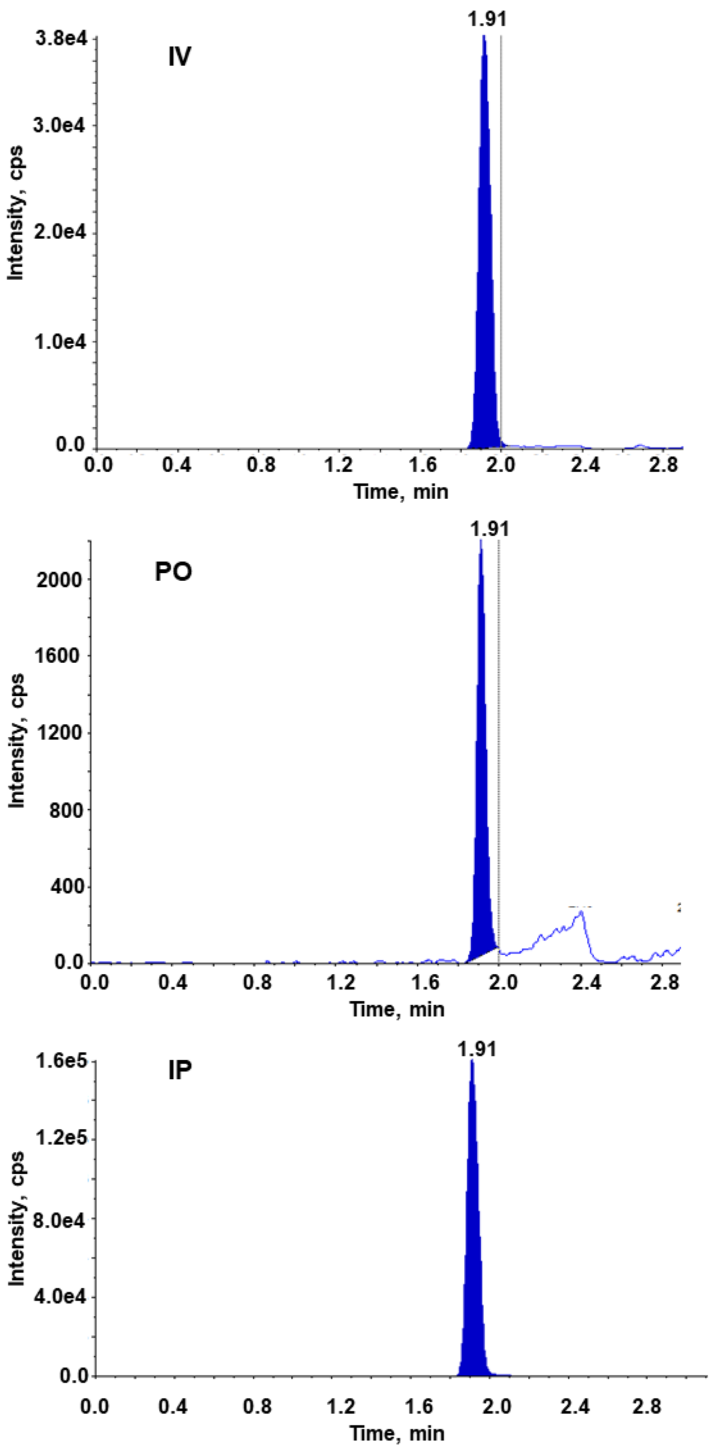

Figure S2C. LC-MS/MS chromatograms of plasma samples obtained after VNPP433-3β administration from IV (0.083 h), PO (0.25 h) or IP (0.25 h) to CD-1 mice.

Figure S2. LC-MS/MS chromatograms of plasma samples obtained after Gal, VNPP414 or VNPP433-3β administration from IV (0.038 h), PO (0.25 h) or IP (0.25 h) to CD-1 mice.

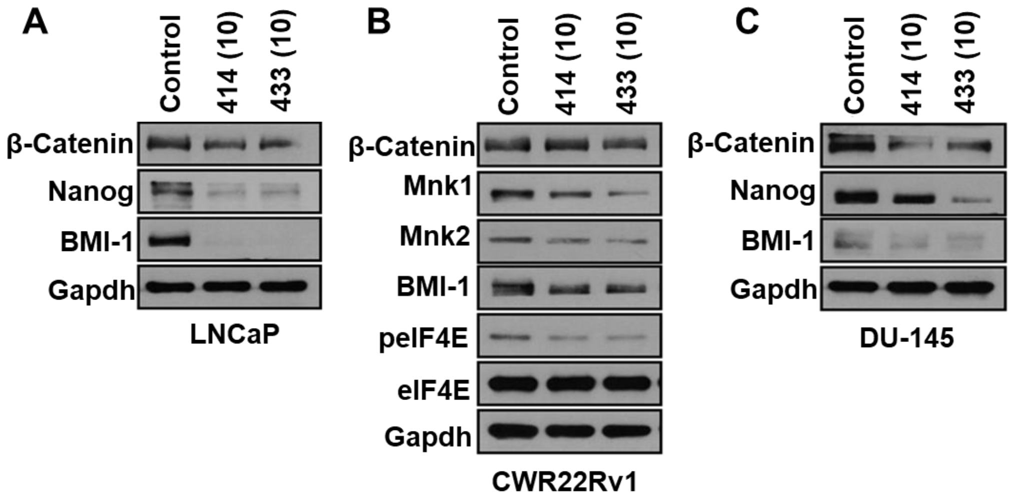

Figures S3A–C. NGGAs modulate EMT markers. (**A**) VNPP414 (414) and VNPP433-3β (433) decreased expressions of β-catenin, Nanog and BMI-1 in LNCaP cells. (**B**) VNPP414 (414) and VNPP433-3β (433) decreased expressions of β-catenin, BMI-1, Mnk1/2 and peIF4E in CWR22Rv1 cells. (**C**) VNPP414 and VNPP433-3β decreased expressions of β-catenin, Nanog and BMI-1 in DU-145 cells.

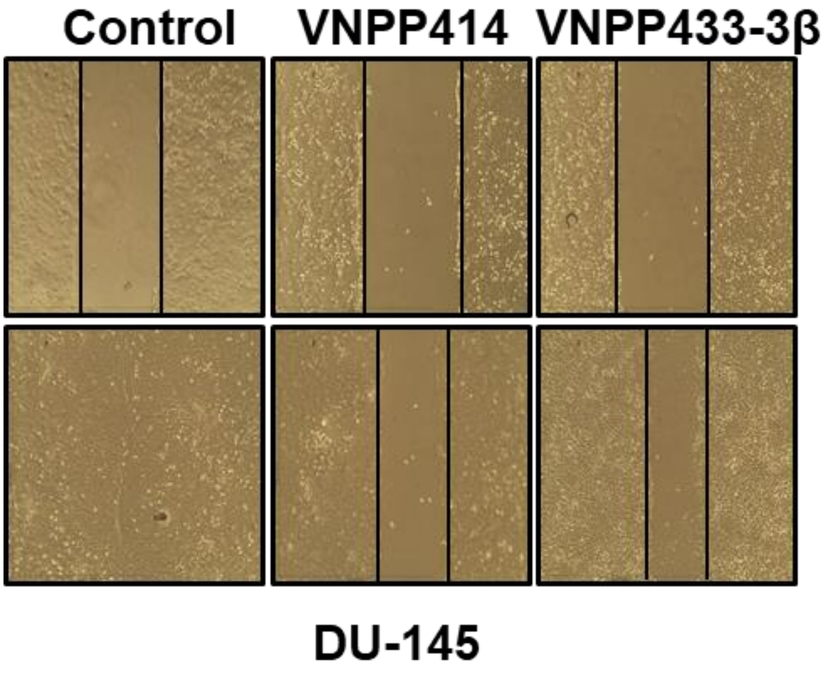

Figure S4. VNPP414 and VNPP433-3β inhibit DU-145 cells migration.

© 2019 by the authors. Submitted for possible open access publication under the terms and conditions of the Creative Commons Attribution (CC BY) license (http://creativecommons.org/licenses/by/4.0/).
